# Salvianolic Acid B Attenuates Toxin-Induced Neuronal Damage via Nrf2-Dependent Glial Cells-Mediated Protective Activity in Parkinson’s Disease Models

**DOI:** 10.1371/journal.pone.0101668

**Published:** 2014-07-03

**Authors:** Jie Zhou, Xiao-Dong Qu, Zhi-Yun Li, Wei-Ji, Qi Liu, Yi-Hui Ma, Jiao-Jiang He

**Affiliations:** Department of Neurosurgery, Lanzhou General Hospital, Lanzhou Command of PLA, Lanzhou, Gansu, China; University of Nebraska Medical Center, United States of America

## Abstract

Salvianolic acid B (SalB), a bioactive compound isolated from the plant-derived medicinal herb Danshen, has been shown to exert various anti-oxidative and anti-inflammatory activities in several neurological disorders. In this study, we sought to investigate the potential protective effects and associated molecular mechanisms of SalB in Parkinson’s disease (PD) models. To determine the neuroprotective effects of SalB *in vitro*, MPP^+^- or lipopolysaccharide (LPS)-induced neuronal injury was achieved using primary cultures with different compositions of neurons, microglia and astrocytes. Our results showed that SalB reduced both LPS- and MPP^+^-induced toxicity of dopamine neurons in a dose-dependent manner. Additionally, SalB treatment inhibited the release of microglial pro-inflammatory cytokines and resulted in an increase in the expression and release of glial cell line-derived neurotrophic factor (GDNF) from astrocytes. Western blot analysis illustrated that SalB increased the expression and nuclear translocation of nuclear factor (erythroid-derived 2)-like 2 (Nrf2). The knockdown of Nrf2 using specific small interfering RNA (siRNA) partially reversed the SalB-induced GDNF expression and anti-inflammatory activity. Moreover, SalB treatment significantly attenuated dopaminergic (DA) neuronal loss, inhibited neuroinflammation, increased GDNF expression and improved the neurological function in MPTP-treated mice. Collectively, these findings demonstrated that SalB protects DA neurons by an Nrf-2 -mediated dual action: reducing microglia activation-mediated neuroinflammation and inducing astrocyte activation-dependent GDNF expression. Importantly the present study also highlights critical roles of glial cells as targets for developing new strategies to alter the progression of neurodegenerative disorders.

## Introduction

Parkinson’s disease (PD) is a chronic progressive degenerative disorder of the central nervous system (CNS) that has the highest incidence in the elderly, with most cases occurring after the age of 50. It affects approximately 6 million people worldwide and the prevalence will double within the next two decades, in parallel with an increasing aged population [1]. PD is characterized by the selective degeneration of mesencephalic dopaminergic (DA) neurons in the substancia nigra pars compacta (SNpc), leading to a loss of DA afferents in the target structure of the striatum and putamen and several motor symptoms, such as bradykinesia, resting tremor, muscle rigidity, and postural abnormalities [2], [3]. Despite dramatic improvements in the clinical treatment of PD, current therapeutic strategies, including DA replacement therapy, can only provide temporary relief of motor symptoms but do not prevent the progressive degeneration and loss of DA neurons [4].

Previous studies on neuroprotection against PD largely focused on the loss of DA neurons; however, increasing evidence supports the important roles of the neuroglia cells in PD-related neuronal injury and functional deficits [5]. Neuroglia cells not only function as the physical support for neurons but also regulate the internal environment of the brain, assist in synaptic connections, control breathing through pH-dependent ATP release and nutrify neurons [6], [7]. Neurodegenerative diseases disrupt the connectivity within brain circuits, which are formed by neuronal-neuronal, neuronal-glial and glial–glial contacts. In addition, neurodegeneration triggers universal and conserved astroglial reactions, which regulate synaptic transmission and the protective/toxic balance, as well as microglia activation, which controls the secretion of inflammatory cytokines [5], [8], [9]. All of these results allow us to regard neurodegenerative diseases as primarily gliodegenerative processes, and some pharmacological agents targeting neuroglia reactions have demonstrated to have neuroprotective effects in laboratory studies [10], [11].

Danshen, a well-known traditional Chinese medical herb, is the dry root and rhizome of *Salvia miltiorrhiza* Bunge. It has been widely used for thousands of years in oriental medicine to treat a variety of diseases, and is also among the most promising drugs in the Chinese drug research field in recent years [12]. The Danshen extract contains more than 18 chemical composites with extensive biological activities, including nonpolar (lipid-soluble) diterpenoidal compounds and water-soluble (hydrophilic) phenolic compounds, among which salvianolic acid B (SalB) is the most abundant component and accounts for its most therapeutic activities [12], [13]. Previous studies have shown that SalB exert anti-cancer activity in human cancer cell lines, such as glioma U87 cells, as well as head and neck squamous cell carcinoma [14], [15]. The therapeutic potential of SalB on hepatic protection, cardiovascular protection, and neural protection has also been proposed in recent studies [16]–[18]. Moreover, SalB was reported to protect human SH-SY5Y neuroblastoma cells against 1-methyl-4-phenylpyridinium (MPP^+^)- or 6-hydroxydopamine (6-OHDA)-induced apoptosis [19], [20]. However, the protective activity of SalB in primary cultured mesencephalic cells and in *in vivo* PD models has not previously been determined. In the present study, the potential neuroprotective activities of SalB on MPP^+^- and lipopolysaccharide (LPS)-induced neuronal injury in primary cultured mesencephalic cells were evaluated. We also investigated the role of neuroglia cells in SalB-induced protective effects and the potential underlying molecular mechanisms, with a particular focus on the nuclear factor (erythroid-derived 2)-like 2 (Nrf2) pathway.

## Materials and Methods

### Mesencephalic cell culture and treatment

**Figure 1:**
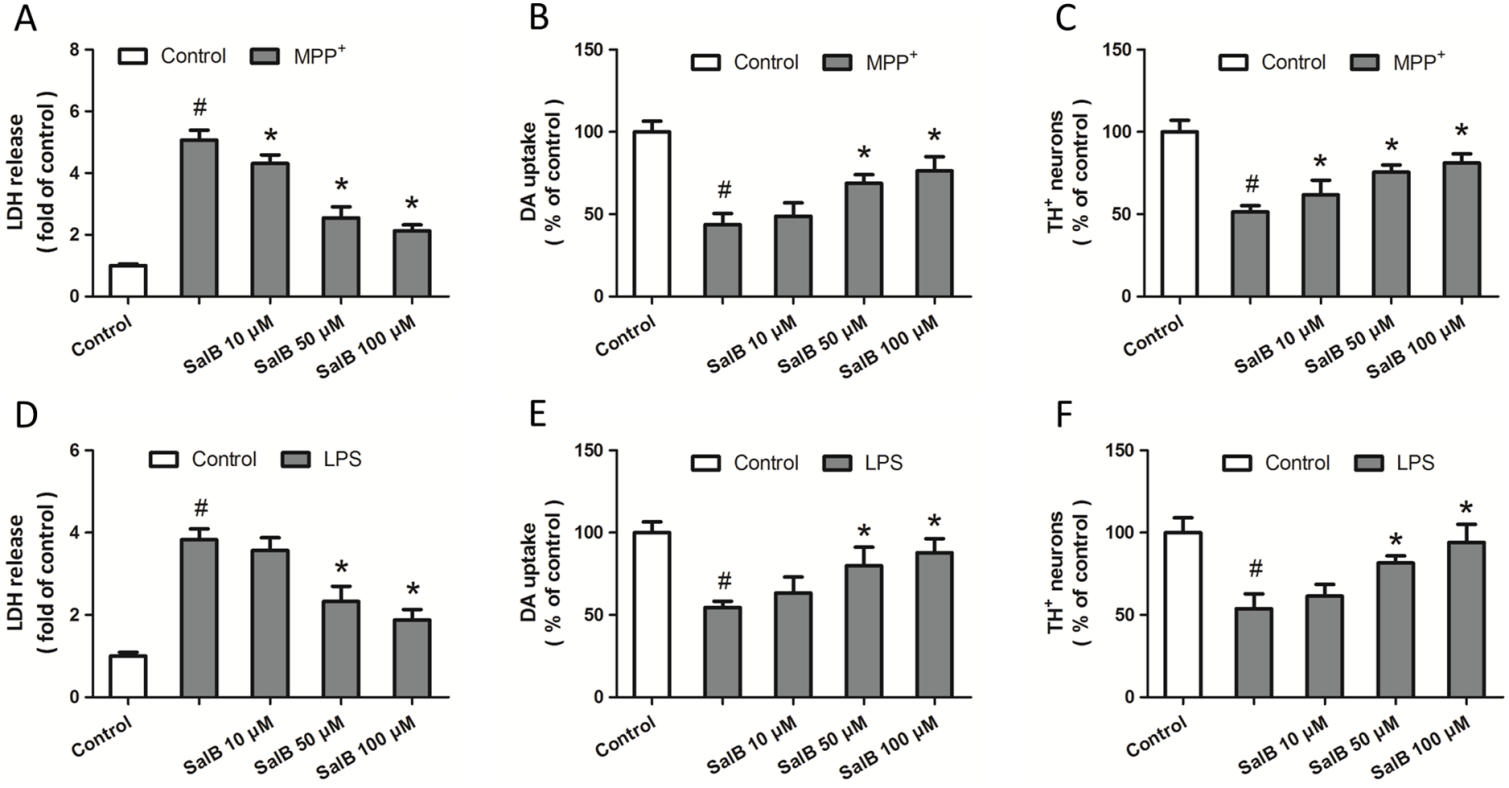
SalB protects DA neurons from toxin-induced toxicity. Mouse midbrain neuron-glia cultures were pretreated with various concentrations of SalB (10, 50 or 100 µM) 2 h prior to stimulation with MPP^+^ (200 µM for 48 h). The cytotoxicity was measured by LDH release assay (A), and the functional status of DA neurons was quanitified by [^3^H] DA uptake assay (B). The number of TH^+^ neurons is expressed as % of control (C). Mouse midbrain neuron-glia cultures were pretreated with various concentrations of SalB (10, 50 or 100 µM) 2 h prior to stimulation with LPS (20 ng/ml for 4 d). The cytotoxicity was measured by LDH release assay (D), and the functional status of DA neurons was quanitified by [^3^H] DA uptake assay (E). The number of TH^+^ neurons is expressed as % of control (F). Data are shown as mean ± SD of five experiments. ^#^ *p*<0.05 vs. Control. *p<0.05 vs. MPP^+^ or LPS.

**Figure 2:**
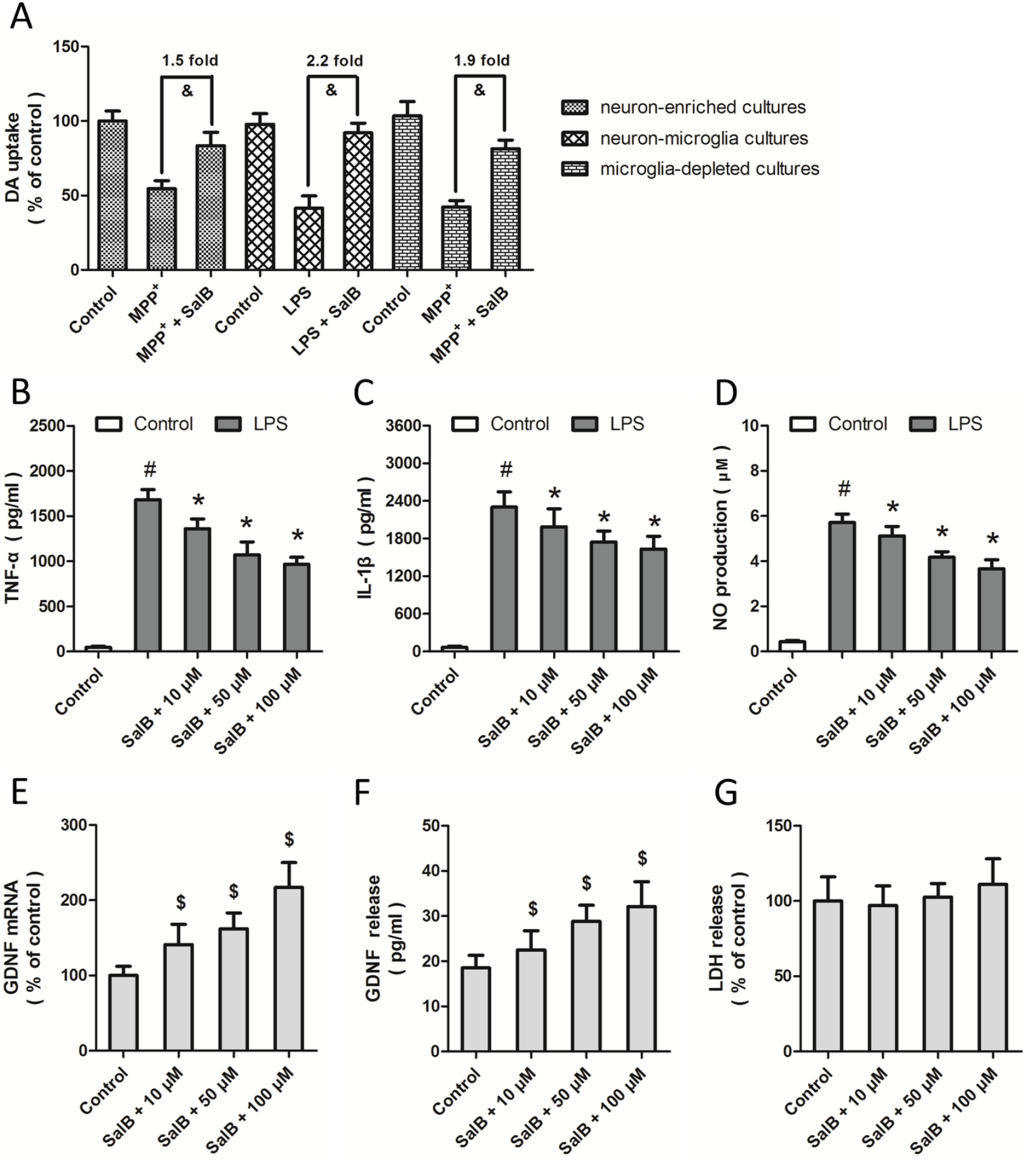
Glial cells mediate the neuroprotective effects of SalB. SalB (100 µM) was applied 2 h prior to stimulation with MPP^+^ (200 µM for 48 h) or 2 h prior to stimulation with LPS (20 ng/ml for 4 d). Neuron-enriched culture, microglia-depleted culture and reconstituted neuron-microglia coculture were used, and the functional status of DA neurons was quanitified by [^3^H] DA uptake assay (A). Microglia-enriched cultures were pretreated with various concentrations of SalB (10, 50 or 100 µM) 2 h prior to stimulation with LPS (20 ng/ml for 4 d). TNF-α (B) and IL-1β (C) were measured by ELISA kit, and NO production was measured using Griess reagent (D). Astroglia-enriched cultures were treated with various concentrations of SalB (10, 50 or 100 µM) for 24 h, and the expression of GDNF mRNA was measured by RT-PCR (E). GDNF release (F) and LDH release (G) were assayed. Data are shown as mean ± SD of five experiments. ^#^ *p*<0.05 vs. Control. *p<0.05 vs. LPS. ^&^ *p*<0.05. ^$^ *p*<0.05 vs. Control.

**Figure 3:**
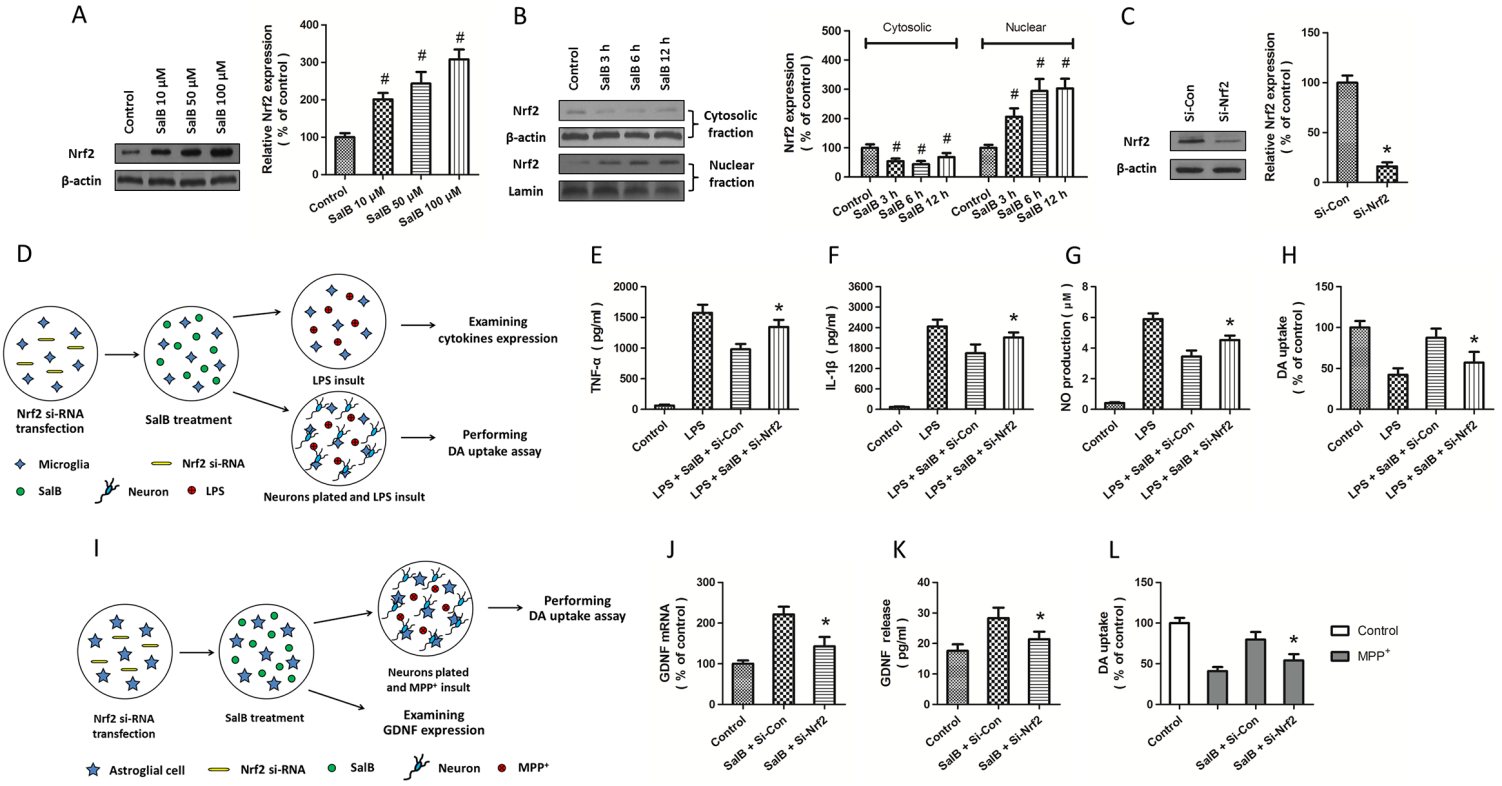
Role of Nrf2 in SalB-induced neuroprotection. Mouse midbrain neuron-glia cultures were treated with various concentrations of SalB (10, 50 or 100 µM) for 24 h, and the expression of Nrf2 was examined by western blot (A). Mouse midbrain neuron-glia cultures were treated with SalB (100 µM), and the expression of Nrf2 in cytosolic fraction and nuclear fraction were examined by western blot at 3, 6 or 12 h after SalB treatment (B). Mouse midbrain neuron-glia cultures were transfected with Nrf2-specific siRNA (Si-Nrf2) or control siRNA (Si-Con) for 72 h, and the expression of Nrf2 was examined by western blot (C). D: Schematic illustration of the experimental protocol (E–H). Microglia-enriched cultures were transfected with Nrf2-specific siRNA (Si-Nrf2) or control siRNA (Si-Con) 72 h prior to SalB (100 µM) treatment and LPS injury (20 ng/ml for 4 d) with (H) or without (E, F and G) neurons plantation. TNF-α (E), IL-1β (F), NO production (G) and DA uptake (H) were assayed. I: Schematic illustration of the experimental protocol (J–L). Astroglia-enriched cultures were transfected with Nrf2-specific siRNA (Si-Nrf2) or control siRNA (Si-Con) 72 h prior to SalB (100 µM) treatment with (L) or without (J and K) neurons plantation and MPP^+^ injury (200 µM for 48 h). The expression of GDNF mRNA (J), GDNF release (K) and DA uptake (L) were measured. Data are either representative of three similar experiments or are shown as mean ± SD of five experiments. ^#^ *p*<0.05 vs. Control. *p<0.05 vs. control siRNA.

All experimental protocols and animal handling procedures were performed in accordance with the National Institutes of Health (NIH) guidelines for the use of experimental animals and approved by the Institutional Animal Care and Use Committee of the Fourth Military Medical University. The mesencephalic neuron-glia cultures were cultured from C57BL6/J mice using a modified method reported by Ossola et al. [11]. In brief, mesencephalic tissues were dissected from embryos at 15–16 days, stripped of the meninges and blood vessels, and minced. The tissues were dissociated by 0.1% trypsin digestion for 15 min at 37°C and gentle trituration. The cells were suspended in minimum essential medium (MEM) supplemented with 10% heat-inactivated fetal bovine serum (FBS), 1 g/L glucose, 2 mM L-glutamine, 1 mM sodium pyruvate, and 50 U/mL penicillin/streptomycin, and plated at a density of 3×10^5^ cells/cm^2^. Before seeding, culture vessels, consisting of 96-well plates, 1.5 cm glass slides or 6 cm dishes were coated with poly-D-lysine (PLL, 50 µg/ml) at room temperature overnight. It was reported that the composition of these neuron-glia cultures was approximately 11% microglia, 48% astroglia, and 41% neurons of which 2.8–3.8% of the cells were TH-positive DA neurons [21]. The neuron-enriched cultures were prepared by adding 10 mM Ara-C to mesencephalic neuron-glia cultures for 48 h at 72 h after seeding. The microglia-depleted cultures were prepared by adding 1.5 mM LME, a microglia toxin, to the mesencephalic neuron-glia cultures 48 h after seeding. The microglia-enriched cultures were prepared from the whole brains of 1–3-day-old mouse pups using an earlier described protocol [21]. Two weeks after seeding, microglia were shaken off for 30 min at 180 rpm, resulting in purity greater than 98%. The astrocytes-enriched cultures were prepared by triturating the encephalon of 1–3-day-old mouse pups in maintenance medium. After 6 days of seeding the medium was replaced with treatment medium for additional 24 h to let the culture stabilize with the low serum medium. Afterwards, to reduce any possible cell perturbation, we added amantadine at a final concentration of 30 µM, resulting in the purity of astrocytes greater than 90%. The reconstituted neuron-microglia cultures were prepared by adding 10 mM Ara-C to the mesencephalic neuron-glia cultures 55 h after seeding. The cultures were incubated with Ara-C for 60 h after which we added 7.5×10^5^ cells/well of the microglia-enriched culture suspended in maintenance medium. The mesencephalic neuron-glia cultures were used in Fig. 1. To investigate the involvement of glial cells in SalB-induced protection, the neuron-enriched cultures, the neuron-microglia cultures and the microglia-depleted cultures were used in experiments as shown in Fig. 2A. The microglia-enriched cultures were used in experiments as shown in Fig. 3D, and the astrocytes-enriched cultures were used in experiments as shown in Fig. 2E–2G and Fig. 3I. The neuron-microglia cultures were used in experiments as shown in Fig. 2B–2D. The neuron-glia cultures were used in all the other experiments.

### LDH release assay

Neuronal cytotoxicity was determined by the release of LDH, a marker of membrane integrity. LDH release into the culture medium was detected using a diagnostic kit according to the manufacturer’s instructions (Jiancheng Bioengineering Institute, Nanjing, China). Briefly, 50 µl of the supernatant from each well was collected to assay the LDH release. The samples were incubated with the reduced form of nicotinamide-adenine dinucleotide (NADH) and pyruvate for 15 min at 37°C, and the reaction was stopped by adding 0.4 mol/L NaOH. The activity of LDH was calculated from the absorbance at 440 nm, and the background absorbance from the culture medium that was not used for any cell cultures was subtracted from all of the absorbance measurements.

### [^3^H] DA uptake assay

The [^3^H] DA uptake assay was performed as described previously [22]. The cultured neurons were incubated with 50 nM [^3^H] for 15 min, and the uptake was stopped by removing the reaction mixture containing the radioligand. The blank values were obtained by incubating the neurons at 0°C, a condition that blocked the specific uptake *in vitro*. The radioactive DA was determined by liquid scintillation counting using a Beckman Tricarb 2900 TR liquid scintillation counter (Fullerton, CA, USA). The nonspecific DA uptake was determined using mazindol (10 mM) and subtracted.

### Immunocytochemistry

After fixation with 4% paraformaldehyde for 15 min at room temperature, cells were washed with NaCl/Pi, permeabilized with 0.2% Triton X-100, and incubated with the primary antibody (anti-TH, 1∶300) overnight at 4°C. The cells were then incubated with the Alexa 488-conjugated secondary antibodies for 2 h at 37°C, and Hoechst 33342 (10 µg/ml) was used to stain the nuclei. The images were captured with an Olympus FV10i Confocal Microscope (Tokyo, Japan). All of the images of one experiment were acquired with the same exposure time to allow comparisons of the relative levels of immunoreactivity between the different treatment conditions. The number of TH-positive cells (at least six images of each group) was counted by an evaluator blinded to the experimental conditions, and the results were represented as a percentage of the control.

### Enzyme-linked immunosorbent assay (ELISA)

To detect the expression of inflammation-related cytokines, the cells were washed with ice-cold phosphate buffered saline (PBS) three times and lysed with a lysis buffer containing protease inhibitors. The amount of TNF-α and IL-1β after various treatments was measured with the sandwich ELISA techniques. Equal amounts of protein (100 µg) from each sample were placed in ELISA kit strips coated with the appropriate Ab. The sandwich ELISA was then performed according to the manufacturer’s instructions (BioSource International, Camarillo, CA).

### Nitrite assay

The production of NO was determined by measuring the accumulated level of its stable metabolite, nitrite, in the supernatant with the Griess reagent (1% sulfanilamide, 2.5% H_3_PO_4_, 0.1% N-(1-naphthyl) ethylenediamine dihydrochloride) as described previously [23].

### Real-Time RT-PCR

RNA was isolated from primary cultured mesencephalic cells using Trizol reagent (Invitrogen, CA, USA). After the equalization of the RNA quantity in each group, reverse-transcription and real time PCR were performed using a commercial available kit (TaKaRa, Dalian, China). Following cDNA generation, quantitative PCR was completed with the Bio-Rad iQ5 Gradient Real-Time PCR system (Bio-Rad Laboratories, Beijing, China). The following primers were used for all of the PCR experiments: GDNF, 5′-GAGAGAGGAACCGGCAAGCT-3′ (forward), 5′-GTTAAGACG -CACCCCCGATT-3′ (reverse); GAPDH, 5′-CCTGGAGAAACCTGCCAAGTAT-3′ (forward), 5′-AGCCCAGGATGCCCTTTAGT-3′ (reverse). The PCR conditions were 94°C (30 s), 58°C (30 s) and 72°C (30 s) for 50 cycles. The amount of GDNF gene expression was normalized to GAPDH, and the values from the control group were set as 100%.

### Short interfering RNA (siRNA) and transfection

Nrf2 siRNA (sc-37049, Santa Cruz, CA, USA) and control siRNA (sc-37007, Santa Cruz, CA, USA) were dissolved separately in Optimem I (Invitrogen, CA, USA). After 10 min of equilibration at room temperature, each RNA solution was combined with the respective volume of the Lipofectamine 2000 solution (Invitrogen, CA, USA), mixed gently and allowed to form siRNA liposomes for 20 min. The primary cultured mesencephalic cells were transfected with the transfection mixture in antibiotic-free cell culture medium for 72 h before MPP^+^ or LPS treatment, and subjected to various measurements.

### Nuclear protein extraction

Nucleus fractions were extracted from primary cultured mesencephalic cells using a multistep extraction protocol. The cells were washed in PBS followed by incubation in a hypotonic buffer (5 mM NaCl, 1 mM MgCl_2_, 1 mM DTT, 10 mM MOPS pH 7.4) for 10 min. Next, one suspension was extracted for the total protein, and the other was treated with a nuclear protein extraction kit (Beyotime Biotechnology, Wuhan, China) and centrifuged at 3400 r.p.m. for 10 min at 4°C. The total and nuclear components were then subjected to western blotting. All incubation steps were carried out at 4°C with ice-cold buffers.

### Western blot analysis

Equivalent amounts of protein were loaded and separated by 10% SDS-PAGE gels, and transferred to polyvinylidene difluoride (PVDF) membranes. The membranes were blocked with 5% nonfat milk solution in tris-buffered saline with 0.1% Triton X-100 (TBST) for 1 h, and then incubated overnight at 4°C with the primary Nrf2 antibody (1∶800), Lamin antibody (1∶1000) or β-actin (1∶600) antibody dilutions in TBST. Next, the membranes were washed and incubated with a secondary antibody for 1 h at room temperature. The immunoreactivity was detected with Super Signal West Pico Chemiluminescent Substrate (Thermo Scientific, Rockford, IL, USA). The analysis software Image J (Scion Corporation) was used to quantify the optical density of each band.

### Animals and treatment

**Figure 4:**
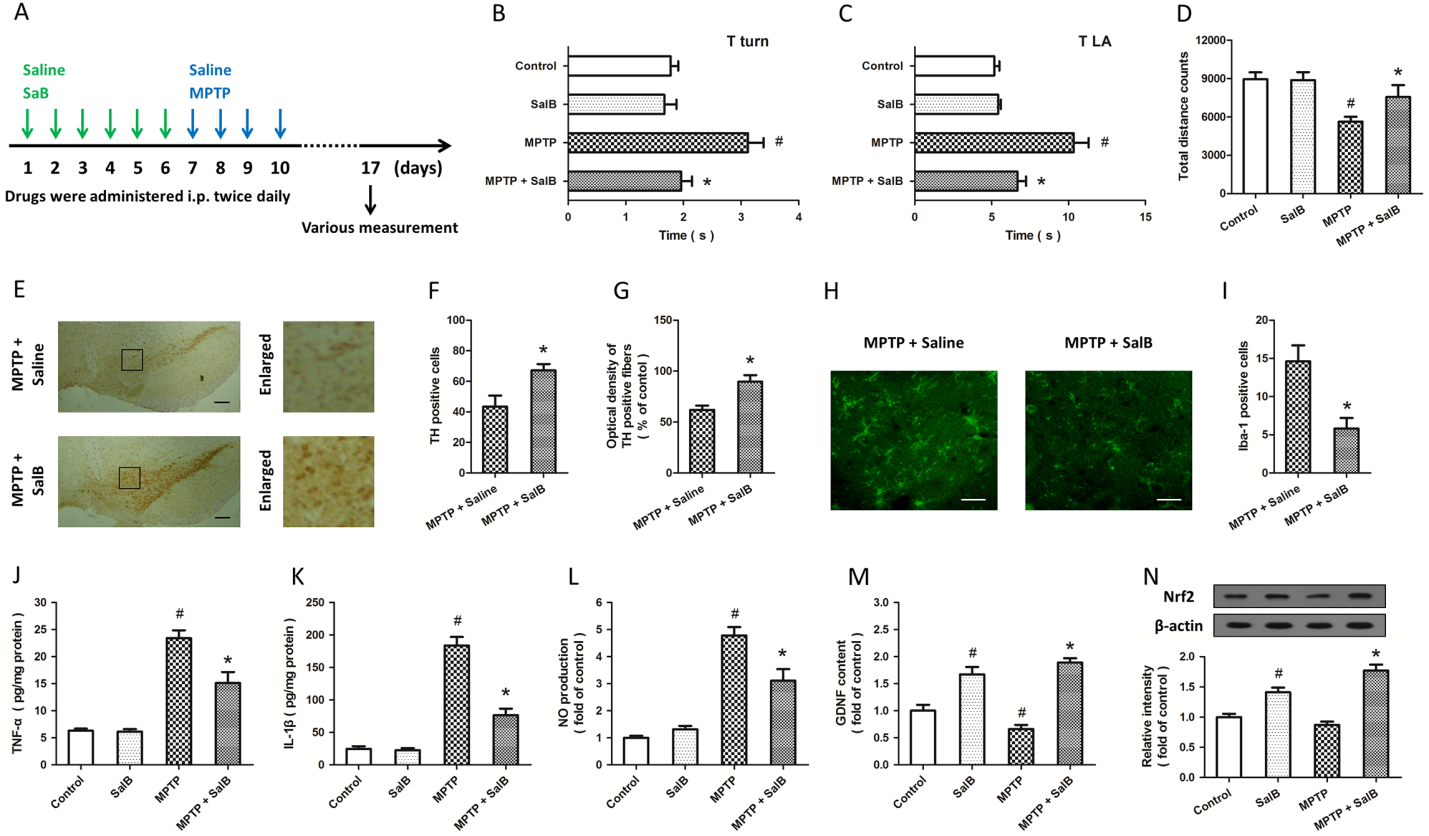
SalB protects against MPTP toxicity in a mouse PD model. A: Schematic of drug administration schedule. Mice were pretreated with SalB (25 mg/kg for 6 d), and injured by repeated administration of MPTP (20 mg/kg for 4 d). Tturn (B) and TLA (C) were defined as the time taken for animals to turn completely downward at the top of an iron pole and for them to arrive at the floor, respectively. Spontaneous locomotor activity was measured by an Opto-Varimex-Minor activity meter (D). Dopaminergic neurons were visualized with TH immunostaining (E). The number of TH-positive neurons (F) and optical density of TH-positive fibers (G) were measured. The activation of microglia was detected by Iba-1 immunostaining (H), and the number of Iba-1-positive cells was counted (I). The expression of TNF-α (J) and IL-1β (K), NO production (L) and GDNF content (M) were examined. The expression of Nrf2 was examined by western blot (N). Scale bars: 200 µm in E and 50 µm in H. Data are either representative of three similar experiments or are shown as mean ± SD of five experiments. ^#^ *p*<0.05 vs. Control. *p<0.05 vs. MPTP.

We used male C57BL/6J mice (3-month old, 25–28 g). The animals had continuous access to food and water and were housed in cages in a room maintained at 20–22°C with a 12 h light/12 h dark cycle. All experimental protocols and animal handling procedures were performed in accordance with the National Institutes of Health (NIH) guidelines for the use of experimental animals (NIH Publications No. 80-23, revised 1996). All efforts were made to minimize the animal numbers and their suffering. The pharmacological effects of SalB in an *in vivo* PD model were examined using the following groups: control, saline treatment alone; SalB, SalB treatment alone; MPTP, treatment of saline followed by 20 mg/kg MPTP; and MPTP + SalB, treatment of 50 mg/kg SalB followed by 20 mg/kg MPTP. SalB (purity >98%) was purchased from the National Institute for the Control of Pharmaceutical and Biological Products (Beijing, China) and dissolved in saline. SalB was administered intraperitoneally twice a day for 6 days before MPTP treatment for 4 days, as shown schematically in Fig. 4A.

### The quantitative measurement of bradykinesia

The pole test was used to determine the degree of bradykinesia at 7 d after MPTP cessation as described previously [24]. Briefly, mice were positioned head upward near the top of a rough-surfaced iron pole (10 mm in diameter and 55 cm in height). The time taken until they turned completely downward (defined as turn time, T turn) and the time taken to arrive at the floor (locomotor activity time, T LA) were recorded. This test was carried out five times in succession for each mouse after one successful practice test.

### The locomotor activity assay

Spontaneous locomotor activity was measured by the Opto-Varimex-Minor activity meter as describe previously [25]. The locomotor activity associated with ambulatory locomotion was defined as the total distance traveled in 1 h. A habituation period of 1 h to the conditions of the experimental room was performed before the experimental procedures. After acclimatization to the experimental room, mice in each group were tested in a dimly lit sound-controlled room. The mice were removed from their home cages and placed into the cage activity system. The data were recorded by the data collection system and the spontaneous locomotor activity was recorded for 1 h.

### Immunohistochemistry

To detect TH-positive DA neurons, serial 30 µm thick coronal sections were cut on a freezing microtome (Leica, Nussloch, Germany). The brain tissue sections were pre-treated with 1% hydrogen peroxide for 15 min and incubated with a rabbit anti-TH antibody (1∶200) overnight at 25°C in the presence of 0.3% Triton X-100 and normal goat serum. The peroxidase activity was visualized by incubating the sections with DAB in 0.05 M tris-buffered saline (pH = 7.6). After several rinses with PBS, the samples were mounted on gelatin-coated slices, dehydrated, and coverslipped in histomount medium. For the Iba-1 immunofluorescence staining, the brain tissue sections were incubated with a rabbit anti-Iba-1 antibody (1∶200), and Alexa 488 donkey-anti-goat IgG (Invitrogen, 1∶300) was used as the secondary antibody for 2 h of incubation. The results were evaluated independently by two specialized neuropathologists who were blinded to all of the experimental groups. The number of TH-positive cells was counted in SNpc at 40×magnification, and the optical density of TH-positive fibers in the striatum (ST) was measured at 100×magnification.

### Statistical analysis

The statistical analysis was performed using SPSS 16.0, a statistical software package. The statistical evaluation of the data was performed by one-way analysis of variance (ANOVA) followed by Bonferroni’s multiple comparisons or unpaired *t* test (two groups). A value of *p*<0.05 was considered statistically significant.

## Results

### SalB protects DA neurons from toxin-induced toxicity

To investigate the potential neuroprotective effects of SalB against PD-related neuronal injury, primary neuron-glia cultures from mouse midbrains, containing neurons, microglia and astrocytes, were pretreated with various concentrations of SalB (10, 50 or 100 µM) 2 h prior to stimulation with MPP^+^. As shown in Fig. 1A, the results of the LDH release assay showed that MPP^+^ injury induced a significant increase in LDH release, which was attenuated by SalB treatment in a dose-dependent manner. After 48 h of exposure to MPP^+^, the functionality of the DA neurons was assessed using the [H^3^] DA uptake assay, and the results showed that SalB preserved the functionality of the DA neurons after MPP^+^ injury in a dose-dependent manner (Fig. 1B). In addition, SalB exerted a similar degree of protection of DA neurons against MPP^+^-elicited damage, which was indicated by the increased number of immunocytochemically stained TH^+^ cells (Fig. 1C). In addition, SalB at all of the concentrations used in our experiments had not effects on the LDH release, DA uptake and the number of TH^+^ cells (Fig. S1).

To confirm the protective activity of SalB in PD, we investigated the role of SalB in a second *in vitro* model in which inflammatory stress is considered to be the main cause for the DA neuronal loss. Primary neurons-glia cultures were pretreated with various concentrations of SalB (10, 50 or 100 µM) 2 h prior to stimulation with LPS, which can activate glia cells to generate and release pro-inflammatory cytokines. The results showed that 4 days of exposure to LPS resulted in obvious neuronal damage as indicated by the increase of LDH release, the decrease of DA uptake and the reduced number of TH-positive cells (Fig. 1D, 1E and 1F). SalB (50 and 100 µM) significantly inhibited the LDH release (Fig. 1D), attenuated the reduction of DA uptake (Fig. 1E), and increased the number of TH^+^ neurons after LPS injury (Fig. 1F), but 10 µM SalB was not effective compared with LPS-injured neurons without SalB pretreatment (*P*>0.05).

### Glial cells mediate the neuroprotective effects of SalB

To discriminate the cell types that mediate the neuroprotective effects of SalB, we used primary cultures containing different combinations of neurons and glial cells (Fig. 2A). In neuron-enriched cultures, in which the purity of the neurons was approximately 92%, SalB protected the functionality of DA neurons from MPP^+^ insults by 1.5-folds compared to MPP^+^ alone. In neuron-microglia cultures, which contained approximately 15% microglia and 85% neurons, SalB protected the functionality of DA neurons from LPS insults by 2.2-fold. In the microglia-depleted cultures, SalB significantly inhibited MPP^+^-induced toxicity by 1.9-fold. It was shown that the protection induced by SalB in the neuron-microglia cultures or in the microglia-depleted cultures was greater than that found in the neuron-enriched cultures.

To confirm the anti-inflammatory activity of SalB, the neuron-microglia cultures were pretreated with SalB at different concentrations (50 and 100 µM) 2 h before the stimulation with LPS. The expression levels of TNF-α and IL-1β were measured by ELISA kits at 6 h (Fig. 2B and 2C), and NO production was measured using the Griess reagent 24 h later (Fig. 2D). The results showed that SalB pretreatment significantly decreased the increased expression of TNF-α and IL-1β and the LPS-induced NO production, and these effects were observed in a dose-dependent manner. These data suggest that the neuroprotective effects of SalB in microglia-contained cultures were related to its inhibitory activities on pro-inflammatory cytokine release.

To determine whether SalB has an effect on the expression and release of GDNF in astrocytes, primary astrocytes-enriched cultures, containing approximately 90% astrocytes and 10% microglia, were used to measure the expression of GDNF mRNA (Fig. 2E). The results showed that SalB significantly increased the expression of GDNF mRNA 48 h after treatment in a dose-dependent manner. Because GDNF can be released to the extracellular spaces to interact with other cells, we also detected GDNF protein content in the culture medium. As shown in Fig. 2F, SalB also increased the protein levels of GDNF in the culture medium, and this effect was not due to the damage of cellular membranes, which was suggested by the result that SalB alone did not affect the LDH release in astrocyte-enriched cultures (Fig. 2G).

### The role of Nrf2 in SalB-induced neuroprotection

To investigate the potential molecular mechanisms underlying SalB-induced protection, the primary neuron-glia cultures were treated with various concentrations of SalB (10, 50 or 100 µM) for 24 h, and the expression of Nrf2 was detected by western blot analysis (Fig. 3A). The results showed that SalB treatment significantly increased the expression of Nrf2 in a dose-dependent manner. We also found that SalB decreased the expression of Nrf2 in the cytoplasm but increased its expression in the nucleus (Fig. 3B). These effects were observed in a time-dependent manner, indicating that SalB treatment induced nuclear translocation of Nrf2 in neuronal cells. To confirm the involvement of Nrf2 in SalB-induced neuroprotection, specific targeted siRNA (Si-Nrf2) was used to down-regulate the expression of Nrf2. As shown in Fig. 3C, the expression of Nrf2 was decreased to approximately 15% of that in the control siRNA-transfected neuron-glia cultures, and the knockdown efficacy of Si-Nrf2 was also confirmed in microglia and astrocytes (data not shown).

To determine the role of Nrf2 in SalB-induced anti-inflammatory activity, the microglia-enriched cultures were transfected with Si-Nrf2 for 72 h before the SalB treatment and LPS insult (Fig. 3D). After the Si-Nrf2 transfection, SalB treatment and LPS injury, microglia were used to measure the expression of pro-inflammatory cytokines and NO production (Fig. 3E, 3F and 3G). The results showed that the knockdown of Nrf2 partially reversed the inhibitory effects of SalB on the LPS-induced expression of TNF-α, IL-1β and NO production. After the transfection with Si-Nrf2 and SalB treatment, neuron-enriched cultures were plated, and the neuron-microglia co-cultures were stimulated with LPS (Fig. 3D). The results of the DA uptake assay showed that the protection of the functionality in DA neurons induced by SalB was attenuated by Nrf2 knockdown, suggesting that SalB-induced anti-inflammatory activity was partially mediated by Nrf2 activation in microglia.

To further investigate whether the SalB-induced regulation of GDNF expression and release was dependent on the activation of the Nrf2 pathway, the astrocytes-enriched cultures were transfected with the Si-Nrf2 for 72 h before SalB and MPP^+^ treatment (Fig. 3I). In the Nrf2 down-regulated astrocytes, the SalB-induced expression of GDNF mRNA and the GDNF release into culture medium were both reduced (Fig. 3J and 3K). After transfection with Si-Nrf2 and SalB treatment, the neuron-enriched cultures were plated and the neuron-astrocytes co-cultures were incubated with MPP^+^ (Fig. 3I). The SalB-induced neuroprotection was also partially prevented by the down-regulation of Nrf2 in astrocytes (Fig. 3L). Together, these results indicated that the SalB-induced GDNF expression and release were partly mediated by Nrf2 activation in astrocytes, which are also attributed to the SalB-induced protection of the functionality in DA neurons.

### SalB protects against MPTP toxicity in a mouse PD model

To confirm the neuroprotective effects of SalB *in vivo*, drugs were administered according to the schematic shown in Fig. 4A. The time taken until the mice turned completely downward (defined as turn time, T turn) and the time taken to arrive at the floor (locomotor activity time, T LA) were recorded. The values of the T turn (Fig. 4B) and T LA (Fig. 4C) in the MPTP-treated mice were significantly higher than those in control animals, indicating the bradykinesic effect of repeated administration of MPTP. Pretreatment with SalB obviously prevented the MPTP-induced prolonged T turn and T LA values (Fig. 4B and 4C). The spontaneous locomotor activity of mice was also measured by an Opto-Varimex-Minor activity meter system, and the results showed that SalB treatment significantly recovered locomotor activity (approximately 55%) compared with the MPTP-damaged animals (Fig. 4D).

In addition, we also investigated the protective effects of SalB using immunostaining, and representative microphotographs of the TH immunostaining in the SNpc are shown in Fig. 4E. After repeated MPTP administration, the number of TH-immunopositive neurons in the SNpc was decreased by 63%, and the optical density of TH-positive fibers was decreased by 71%. SalB pretreatment (25 mg/kg for 6 days) protected TH-positive neurons and TH-positive fibers against MPTP-induced damage (Fig. 4F and 4G). To investigate the effects of SalB in neuroinflammation after MPTP injury, the activation of microglia was detected by immunostaining with Iba-1 (Fig. 4H). As shown in Fig. 4I, the increased number of Iba-1 positive cells induced by MPTP administration was significantly reduced by SalB treatment, indicating the inhibitory activity of SalB on MPTP-induced microglia activation.

Furthermore, we detected the expression of pro-inflammatory cytokines in our *in vivo* model. MPTP administration significantly increased the expression of TNF-α, IL-1β and NO production in the brain homogenates, and all of these changes were markedly attenuated by SalB treatment (Fig. 4J, 4K and 4L). The content of GDNF in the brain tissues was decreased by MPTP injury, but SalB significantly increased the BDNF content in the presence or absence of MPTP (Fig. 4M), which was consistent with our *in vitro* results (Fig. 2F and 3K). Finally, we detected the expression of Nrf2 by western blot analysis in brain tissues after MPTP insult (Fig. 4N), and the results showed that SalB pretreatment significantly increased the expression of Nrf2 not only in the presence but also in the absence of MPTP treatment, suggesting the involvement of Nrf2 activation in SalB-induced neuroprotection *in vivo*.

## Discussion

The ideal therapeutic strategy for treating patients with PD is to not only increase the striatal dopamine content but also to inhibit further degeneration of the surviving DA neurons in the SNpc of the ventral midbrain [26]. To date, clinical treatments are relied, in most cases, on the elevation of dopamine levels by the use of L-DOPA, its precursor or activating dopaminergic receptors via specific agonists, such as the ergot alkaloid derivatives. All of these drugs fail to prevent the progression of the degenerative process, and are limited by a progressive decrease in drug response, motor fluctuations, dyskinesias and drug-induced toxicity [27]–[29]. Thus, a new focus has shifted onto alternative therapeutic approaches that could provide an independent therapy or offer neuroprotective support to the existing drugs. Several natural products with low toxicity, including active constituents of plants, herbs, and bioactive ingredients from other natural sources, have been investigated in *in vitro* and *in vivo* PD models [30], [31]. The natural product SalB is an active constituent of Danshen, an herb widely used around the world for the treatment of cerebrovascular and cardiovascular disorders [32], [33]. In the present study, we found that SalB treatment significantly attenuated MPP^+^- and LPS-induced neuronal damage in mesencephalic cell cultures, and also preserved the neurological function in an *in vivo* PD model. Among many compounds with neuroprotective activity, SalB has unique advantages, such as an abundance in medical herbs, a low toxicity in human subjects and the ability to cross biological membranes and the blood–brain barrier even in an energy deficient environment [17], [34]. Our results extended the therapeutic potential of SalB to the field of neurodegenerative diseases.

The synthetic heroin analogue 1-methyl-4-phenyl-1,2,3,6-tetrahydropyridine (MPTP) is a neurotoxin that can kill DA neurons, inducing irreversible and severe motor abnormalities almost identical to those observed in PD in humans, non-human primates and rodents, and it is widely used as a tool-drug to investigate PD [35], [36]. The neurotoxic effects of MPTP are due to its active metabolite MPP^+^, an excellent substrate for the dopaminergic transporter, which is formed by the monoamine oxidase (MAO) B-mediated oxidation of MPTP in glia and serotoninergic neurons [37]. MPP^+^ is selectively taken up by dopaminergic terminals via the DA transport system and concentrated in neuronal mitochondria in the SNpc, and the associated mechanisms underlying MPP^+^-induced neuronal injury involves the mitochondrial electron transport chain complex I inhibition, the opening of the mitochondrial transition pore, the influence on intracellular signaling pathways, and glia cells -mediated central inflammation [38], [39]. Several previous studies that investigated potential neuroprotective agents or related molecular mechanisms of PD were performed in neural cell lines, such as PC12 cells, HT22 cells or SH-SY5Y neuroblastoma cells. Furthermore, the protective activity of SalB against MPP^+^ injury was recently demonstrated only in SH-SY5Y neuroblastoma cells [20]. Our present experiments confirmed the protective effects of SalB in primary cultured mesencephalic cells, and also in an *in vivo* mouse PD model. In addition, we also found that SalB significantly increased DA uptake in primary cultured mesencephalic cells, one of the most important physiological functional indicators of DA neurons. All of these results are critical in supporting the use of SalB in further clinical trials to test the protective activity in human subjects, and are of more relevance to clinical therapy.

Although the cause and mechanisms underlying PD are not fully understood, current evidence suggests that the cellular factors involved in the tissue destruction underlying PD are not confined to neurons, and in particular, both astrocytes and microglia appear to play important roles in the initiation and progression of PD [40]. Glial cells are traditionally known as the support cells for neurons in CNS, but recent studies have shown that their roles in neurodegeneration are not only secondary to neuronal dysfunction. Several previous studies have demonstrated that both astrocytes and microglia were activated under PD conditions, and their roles are very dynamic and cell-type dependent [41]–[43]. Astrocytes and microglia may exert harmful effects by producing pro-inflammatory and cytotoxic mediators that kill neurons or form scars that barricade axonal regeneration, but in certain circumstances, these cells can turn into highly protective cells, and produce anti-inflammatory cytokines, express and release a panel of pro-survival, neurotrophic and pro-regenerative factors, thereby facilitating neuronal recovery and repair [44]–[46]. To understand the different cell type interactions and to elucidate the molecular mechanism underlying the neuroprotective effect of SalB, we used an *in vitro* system containing the three main PD-related cell types, namely neurons, microglia, and astrocytes. In reconstituted neuron-microglia cultures, containing only neurons and microglia, the anti-inflammatory activity of SalB was investigated after LPS treatment. In addition, in microglia-depleted cultures, which contained only neurons and astrocytes, the effects of SalB on the expression and release of GDNF after MPP^+^ insult were detected. Furthermore, we also confirmed the relationship between SalB-induced protection against *in vitro* neuronal injury in PD and its effects on the regulation of glia cells through a comparison of the results in neuron-enriched cultures and neuron-glia cultures. Importantly, these co-culture systems allowed us to study the role of glia in SalB’s beneficial effects and their cross talk to neurons, which would be an important supplement to the data obtained from animal experiments.

As the innate immune system for the CNS, microglia comprise approximately 10–20% of adult glia, and can be identified using common macrophage markers, such as Iba-1 [47]. Inflammatory cytokines are recognized to represent important components of glial activation, and among the cytotoxic molecules produced by activated microglia, TNF-α, IL-1β and nitric oxide (NO) from inducible nitric oxide synthase (iNOS) or neuronal nitric oxide synthase (nNOS) represent three key harmful mediators [48], [49]. In the present study, we found that SalB significantly reduced the LPS-induced release of TNF-α, IL-1β and NO in neuron-microglia cultures. Moreover, SalB treatment decreased the number of Iba-1 positive cells in brain tissue sections, and the SalB-induced inhibition of these cytokines was also observed in our *in vivo* PD model. Together these results highlight the direct anti-inflammatory properties of SalB in the neurodegeneration caused by microglial activation, which agree with a recent report suggesting that SalB attenuates the secondary neuronal damage after TBI through regulating neuroinflammation [17]. In addition, our results showed that the protection induced by SalB in microglia-depleted cultures was more prominent than the one observed in neuron-enriched cultures (1.9-fold vs. 1.5-fold), suggesting that the presence of astrocytes increases the protective effects of SalB. Astrocytes function as a support for migrating neuronal precursors in the developing brain and may also function as neuronal precursors in neurodegenerative conditions [50], [51]. Reactive astrogliosis is considered one of the most reliable and sensitive markers of diseased tissue that protects neurons through the synthesis and release of glutathione and growth factors, such as the GDNF family members, which was also demonstrated in our present study [11], [52]. Our results in primary cultured mesencephalic cells and MPTP-treated animals confirmed the effects of SalB on BNDF generation and the release both in the presence and absence of PD related neuronal injury. Therefore, our present data suggest that SalB protects DA neurons by a dual action: reducing microglial activation-mediated neuroinflammation and inducing astrocyte activation-dependent GDNF expression.

Nrf2 (NFE2L2), sometimes referred to as the master regulator of antioxidants and detoxification, is a transcription factor that belongs to the cap and collar family of transcription factors having a distinct basic leucine-zipper motif [53]. Under physiological or unstressed conditions, Nrf2 is kept in the cytosol by a cluster of proteins, such as Kelch-like ECH-associated protein 1 (Keap1) and Cullin 3, which degrade Nrf2 quickly by ubiquitination [54]. When the redox balance is tipped toward the oxidative side, Nrf2 translocates into the nucleus, activates the antioxidant response element (ARE) pathway, and increases the expression of various protective genes, such as heme oxygenase-1 (HO-1), NADPH quinone oxidoreductase (NQO1) and the catalytic and modulatory subunits of g-glutamyl synthase (GCLM, GCLC), etc [55], [56]. Several previous studies have shown that the expression and nuclear localization of Nrf2 were decreased in hippocampal CA1 neurons and surrounding glia in SN in PD brains [57], which was also confirmed in our studies. In *in vitro* cell culture models, increased neuronal Nrf2 activation was reported to protect neurons from oxidative insults induced by parkinsonian neurotoxins including MPP^+^, 6-OHDA, and rotenone [58]. In the present study, we found that the knockdown of Nrf2 expression through specific siRNA transfection in both microglia and astrocytes decreased the protection induced by SalB after MPP^+^ or LPS insults, suggesting that Nrf2 participates in prevention of inflammation in PD by a mechanism that involves microglia and factors secreted by astrocytes, which is in agreement with other studies [59], [60]. During the past few decades, more than 100 bioactive compounds derived from natural products have been demonstrated to be activators of the Nrf2/ARE pathway that can induce Nrf2 to provide favorable effects in experimental models of neurological diseases [61]. A more recent study showed that salvianolic acid A, another aqueous extract of *Salvia miltiorrhiza*, protected retinal pigment epithelial cells against hydrogen peroxide-induced oxidative stress through the activation of Nrf2/HO-1 signaling [62]. Our results demonstrated that SalB increased the expression and nuclear translocation of Nrf2 in the presence and absence of the MPP^+^ insult, and the protection induced by SalB treatment was partially reversed by Nrf2 knockdown. All of these data strongly support that salvianolic acids are natural product-derived pharmacological modulators of the Nrf2/ARE pathway, and they are effective in the treatment of PD-related neuronal injury.

**Figure 5:**
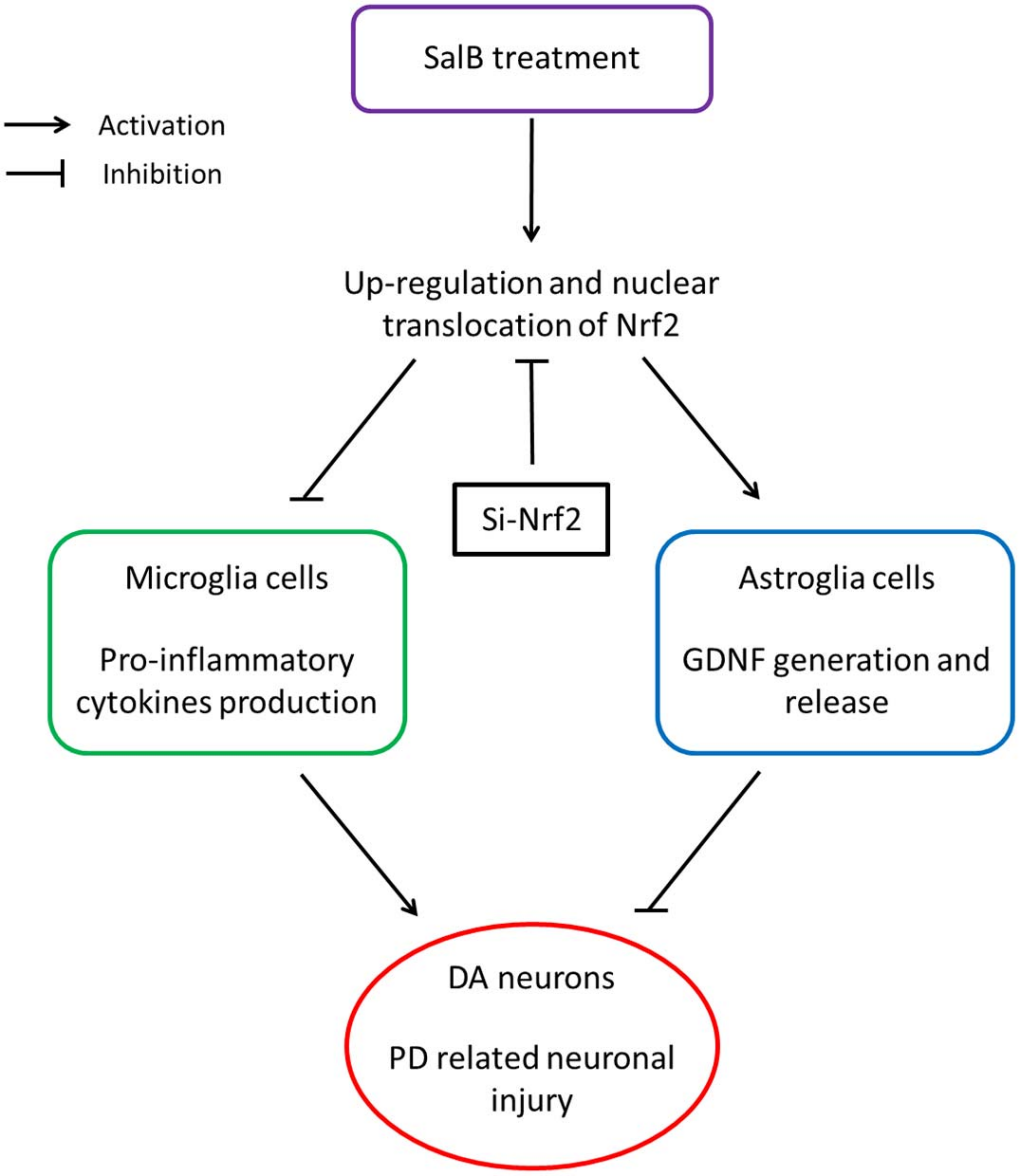
A proposed diagram tying together the various observations involved in SalB-induced neuroprotection against neuronal damage in PD models.

In conclusion, this study demonstrated the neuroprotective effects of SalB in both *in vitro* and *in vivo* PD models. A summary of our findings is shown in Fig. 5. We propose that MPTP administration causes DArgic neurodegneration in the SNpc accompanied by the activation of microglia and astrocytes. SalB treatment increased the expression and nuclear translocation of Nrf2 in both microglia and astrocytes, thereby attenuating the microglia-mediated production of pro-inflammatory cytokines and increasing the astrocyte-dependent generation of GDNF. Importantly, the present study also highlights critical roles of glial cells as targets for developing new strategies to alter the progression of neurodegenerative disorders.

## Supporting Information

Figure S1 SalB used in the experiments has no toxicity. Mouse midbrain neuron-glia cultures were treated with various concentrations of SalB (10, 50 or 100 µM) for 24 h. The cytotoxicity was measured by LDH release assay (A), and the functional status of DA neurons was quanitified by [^3^H] DA uptake assay (B). The number of TH^+^ neurons is expressed as % of control (C). Data are shown as mean ± SD of five experiments.
(TIF)
