# Supplementary figures and images for: Salvianolic Acid B Attenuates Toxin-Induced Neuronal Damage via Nrf2-Dependent Glial Cells-Mediated Protective Activity in Parkinson’s Disease Models
Source: PLoS One. 2014 Jul 3;9(7):e101668. doi: 10.1371/journal.pone.0101668 (PMC4081637; doi:10.1371/journal.pone.0101668)

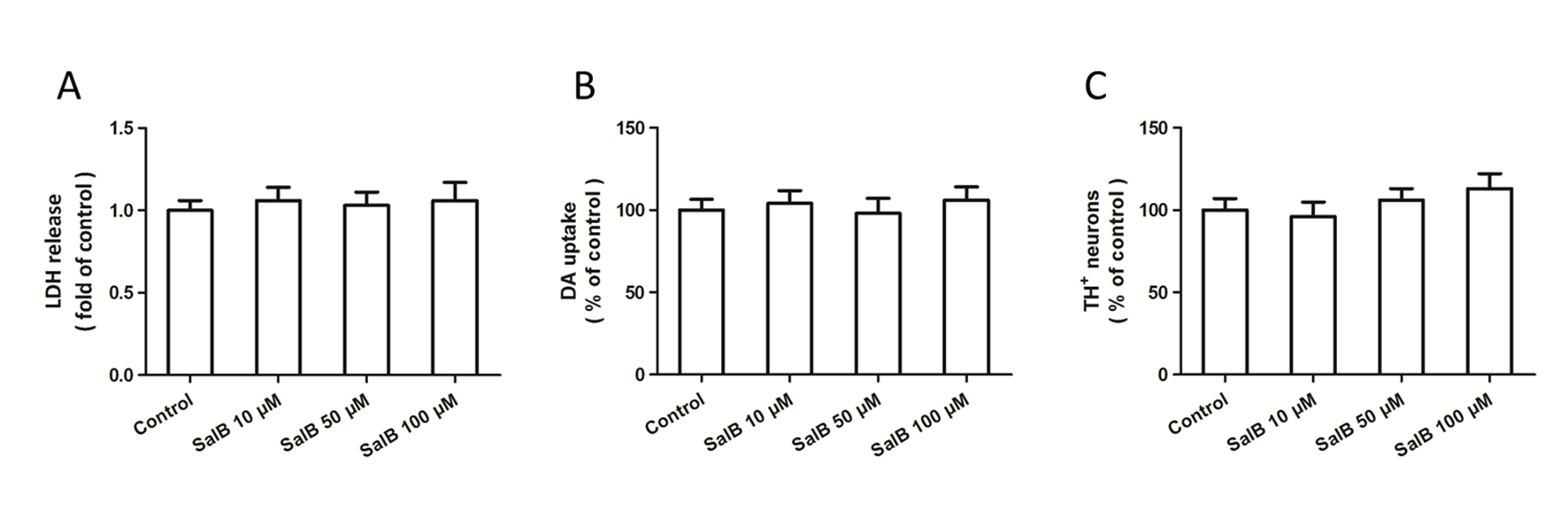

Supplement: Figure S1 — SalB used in the experiments has no toxicity. Mouse midbrain neuron-glia cultures were treated with various concentrations of SalB (10, 50 or 100 µM) for 24 h. The cytotoxicity was measured by LDH release assay (A), and the functional status of DA neurons was quanitified by [3H] DA uptake assay (B). The number of TH+ neurons is expressed as % of control (C). Data are shown as mean ± SD of five experiments. (TIF) [file pone.0101668.s001.tif]
